# ATRA Induced Reactive Hemophagocytosis: a Case Report

**DOI:** 10.4084/MJHID.2011.034

**Published:** 2011-09-08

**Authors:** Monica Sharma, Jasmita Dass, Seema Tyagi

**Affiliations:** Department of Hematology, All India Institute of Medical Sciences, New Delhi

## Abstract

All trans-retinoic acid (ATRA) is a targeted therapy, used in Acute Promyelcytic leukemia (APL) and causes the abnormal promyelocytes to differentiate in to mature leucocytes, however their clearance in vivo is not known. ATRA has been found to be associated with hemophagocytosis, but sometimes one may find phagocytosis of differentiated cells by histiocytes without the overt manifestations of hemophagocytic syndrome. We report a case of APL showing differentiated cells being phagocytosed by marrow histiocytes while patient was getting ATRA therapy.

## Introduction:

All trans-retinoic acid (ATRA) is a targeted therapy, used in Acute Promyelcytic leukemia (APL) to induce complete remission (CR) through differerentiation of abnormal promyelocytes to mature neutrophils, and they are cleared possibly by apoptosis because differentiated hematopoietic cells including mature neutrophils have been observed to undergo apoptosis in vitro^1^ however the mechanism of their clearance in vivo is not known. All the APL patients undergoing differentiation therapy do not demonstrate the phagocytosis by histiocytes in the bone marrow. Secondary hemophagocytic syndrome, a life threatening condition is known to be associated with ATRA syndrome.^2^

We report a case of APL which showed hemophagocytosis with the differentiated cells being phagocytosed by marrow histiocytes while on ATRA therapy. The patient did not have any clinical symptoms suggestive of hemophagocytic syndrome secondary to ATRA therapy.

## Case report:

**Figure 1.**
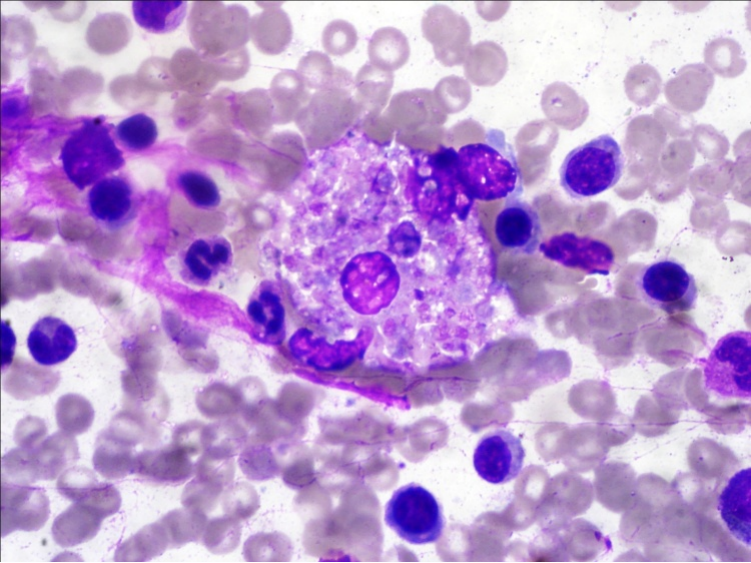
Jenner giemsa stained bone marrow smear showing histiocyte containing engulfed neutrophils in their cytoplasm (1000X).

A 19 years old girl was admitted with complaints of fever for 1 week, menorrhagia and gum bleeding for 5 days. On examination she was pale and had fever. There was no lymphadenopathy or hepatosplenomegaly. She had anemia (hemoglobin 8.2 g/dl), leucocytosis (total leukocyte counts-15x10^9^/L) and thrombocytopenia (platelet counts-15x10^9^/L). The differential counts at this time showed polymorphs 07, lymphocytes 28, Monocytes 03, Blasts 03 and abnormally granulated promyelocytes 59%. Bone marrow examination revealed infiltration by abnormal hypergranular promyelocytes (84%) with multiple auer rods and 02% blasts. Immunofluoresence for anti-PML antibody(PG-M3)^3^ was positive. PML-RARA fusion transcript by RT-PCR was positive for t(15;17). She did not have any feature of disseminated intravascular coagulation. Patient was started on All trans-retinoic acid (ATRA) 45mg/m^2^ in two divided doses along with Daunorubicin 60mg/m^2^ daily for 3 days. She was also given supportive treatment with whole blood and platelet transfusion and antibiotics. Patient had symptomatic improvement and peripheral smear showed myeloid maturation, with complete differentiation on day 24. She did not show any features of ATRA syndrome. Bone marrow repeated at day 34 revealed a remission marrow with 1% blast and disappearance of abnormal promyelocytes. Though there was complete differentiation with disease in remission, there was evidence of phagocytosis, the macrophages were seen with engulfed neutrophillic cell debris (figure 1 **and** 2) which stained positive for MPO.

Patient did not have any fever, joint pains, organomegaly or any other manifestations of macrophage activation syndrome. She did not demonstrated hypertriglyceridemia or hypofibrinogenemia. Patient was continued on ATRA, and after 4 months of treatment she was in complete haematological remission without any complications.

## Discussion:

In APL a high rate of complete remission can be seen with ATRA therapy which induces differentiation of abnormal promyelocytes to neutrophils, however their clearance in vivo is not known. Abormal promyelocytes induced to differentiate towards mature neutrophils possibly die by apoptosis but apoptotic cells are only rarely evident in bone marrow aspirate or trephine sections. ATRA has also been implicated as a cause of secondary hemophagocytic syndrome possibly due to release of pro-inflammatory cytokines during differentiation which activate the macrophages. This has been considered as a manifestation of ATRA syndrome.^2^

It has been observed that human neutrophils derived from peripheral blood or acutely inflamed joints aged in culture undergo morphological changes like nuclear chromatin fragmentation, which are changes of apoptosis. The programmed Cell Death in the neutrophil then leads to its recognition by macrophages.^4^

The macrophages observed in the bone marrow of this case during ATRA therapy suggests a possible mechanism that first recognises & then engulfs the differentiated cells by macrophages. Several recognition mechanisms of intact neutrophils have been described like increased adhesion of promyelocytes to thrombospondin during differentiation induction by ATRA treatment,^5^ also ATRA has been known to up-regulate CD36 expression in human monocytes/macrophages.^4^ There is a suggested possible role of thrombospondin & histiocyte surface molecule CD36 in the recognition & clearance of promyelocytes during the differentiation induction by ATRA *in vivo*. Enhancement of thrombospondin-mediated phagocytosis of neutrophils undergoing apoptosis by proinflammatory cytokines such as GM-CSF, IFN-gamma, IL-1β, TNF-α and TGF-β1 has been reported & modulation of cytokine secretion/expression in abnormal promyelocytes treated by ATRA has also been described. These cytokine-modulations may be responsible for the variation of phagocytosis observed in APL cases.^6^ The elimination of differentiated cells in vivo by histiocytic phagocytosis may be seen in bone marrow on different days post ATRA or during ATRA therapy, suggesting a presence of possible mechanisms that eliminate the abnormal promyelocytes by enhanced expression of some signal on the cell surface before they lose membrane integrity & lyse as the ultimate step of apoptosis.^7^ Kumakura et al have reported two cases with similar observations in the bone marrow on D29 and D17.^8^

Phagocytosis may be an incidental but interesting finding in our case as the patient remained asymptomatic & attained complete morphological remission on ATRA therapy without any clinical consequences.

## Conclusion:

Phagocytosis may be an interesting finding in the bone marrow of the patient treated with ATRA, whose bone marrow before starting therapy was not showing any such changes. It seems to be a normal physiological process by which differentiated cells are cleared from body. Moreover, this case did not show any serious clinical associations or criteria for secondary hemophagocytosis or ATRA syndrome and thus not requiring any a specific treatment or withdrawal of the drug.

## Figures and Tables

**Figure 2.**
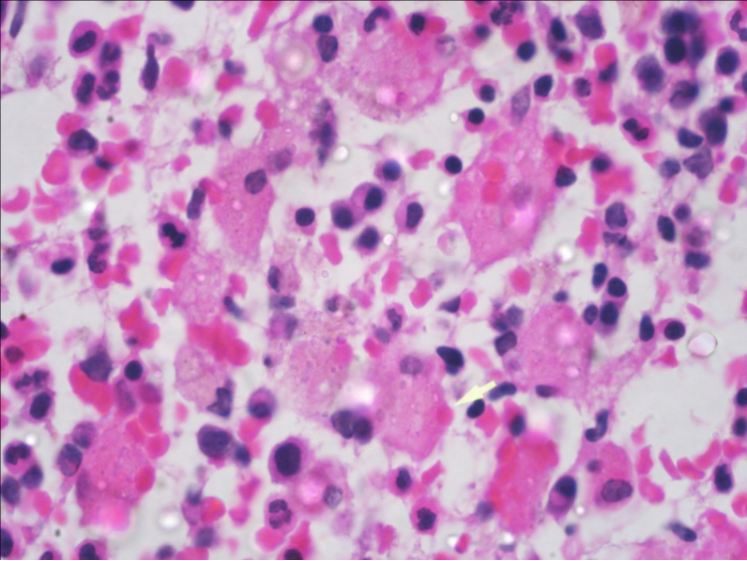
Haematoxylin and Eosin stained bone marrow trephine section showing numerous histiocytes containing neutrophilic debris in the cytoplasm (1000X)
